# Aesthetic compatibility assessment of consolidants for wall paintings by means of multivariate analysis of colorimetric data

**DOI:** 10.1186/s13065-018-0465-7

**Published:** 2018-09-21

**Authors:** Francesca Becherini, Caterina Durante, Elsa Bourguignon, Mario Li Vigni, Vincent Detalle, Adriana Bernardi, Patrizia Tomasin

**Affiliations:** 1 Institute of Atmospheric Sciences and Climate, National Research Council, Corso Stati Uniti 4, 35127 Padua, Italy; 2 Institute of Condensed Matter Chemistry and Technologies for Energy, National Research Council, Corso Stati Uniti 4, 35127 Padua, Italy; 3 Laboratoire de Recherche des Monuments Historiques, 29 rue de Paris, 77420 Champs-sur-Marne, France; 4 ChemSTAMP s.r.l., Via G. Campi 183, 41125 Modena, Italy

**Keywords:** Calcium alkoxides, Consolidation treatment, Color variation, Wall paintings conservation, Mortar, Pigments, Biocide, Quaternary ammonium compounds, PCA, PARAFAC

## Abstract

**Background and methods:**

Wall paintings and architectural surfaces in outdoor environments are exposed to several physical, chemical and biological agents, hence they are often treated with different products to prevent or slow down their deterioration. Among the factors that have to be taken into account in the selection of the most suitable treatment for decorated surfaces, the aesthetic compatibility with the substrate is of great importance in the cultural heritage field; minimizing colour variation after treatment application is a crucial issue in particular for painted surfaces. In the framework of the European Project Nanomatch the color variation induced on wall painting mock-ups by the two innovative consolidants (calcium alkoxides) developed was evaluated using colorimetry in comparison with two traditional products. In this work these innovative consolidants have been also tested in combination with two commercial biocides and the results of colorimetric measurements discussed. Moreover, as the univariate approach didn’t allow to draw clear conclusions on the relation between the different sources of data variability, multivariate analysis was performed on colorimetric data.

**Results:**

Principal Component Analysis and multi-way Parallel Factor Analysis (PARAFAC) were successfully applied to colorimetric data to investigate the short-term effects of the application of different consolidants on wall painting surfaces, making it possible to study at the same time the different sources of data variability, i.e. treatments, painting techniques, pigments. Finally, a ranking list of the treatments under study in terms of colour variation induced on the surface was established, in function of the painting technique and pigment, taking also in consideration the combination consolidant/biocide. In particular, given the true multi-way nature of the data, PARAFAC model turned out to be extremely useful in the study of the dependence of colour variation on pigments, a critical issue for painted surfaces, that was not clear using univariate approach.

**Conclusions:**

Multivariate approach to colorimetric data and especially 3-way PARAFAC method resulted a powerful technique to evaluate in short-term the color compatibility of consolidants for wall paintings, improving data interpretation and visualization, and thus outperforming the univariate statistical analysis.
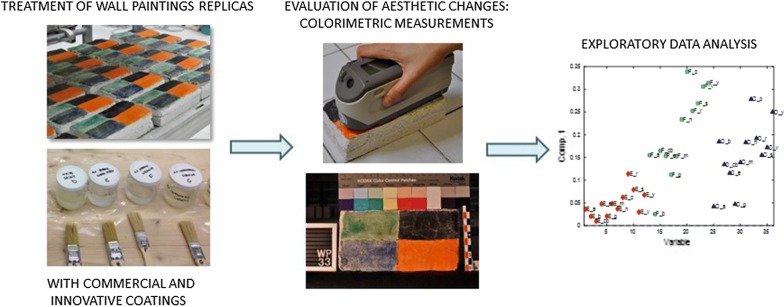

## Introduction

Wall paintings have been cultural expressions of human creativity throughout history, hence their deterioration constitutes a loss affecting a significant part of the world’s Cultural heritage. The conservation of historic decorated surfaces is a difficult issue, due to the great variety of materials and painting techniques, their complex structure and because they are integral to the architectural ensemble [1, 2]. As recommended by the ICOMOS 14th General Assembly, “*all interventions, such as consolidation, cleaning and reintegration, should be kept at a necessary minimal level to avoid any reduction of material and pictorial authenticity*” [3]. In cases of large losses, conservation has to strike a balance between a visually coherent surface and the integrity of the original material. Previous restoration works, some of them considered historic, represent often an additional problem to be faced. A number of organic and inorganic treatments have been widely studied to enhance stone and stone-like materials durability, especially in urban environments, and their advantages and disadvantages have been analyzed in depth [4–6]. As it is well discussed in [7, 8], compatibility is a multifaceted concept, that “*cannot be defined in absolute terms and independently of the case in consideration”.* The compatibility of any product (consolidant, water repellent, etc.) can be related to many different parameters of the substrate and of the product itself [7]. Colour is one of the parameters of the treated material to be taken into consideration and it is particularly important when dealing with paintings, as “*colour changes can alter the entire appearance and perception of a painting*” [9], and consequently the vision and interpretation of the image [10]. Therefore, any treatment application should not result in the alteration of surface appearance, in particular of its colour [11–13].

During the European Project Nanomatch [14] two calcium alkoxides, (Ca(OTHF)_2_ and Ca(OEt)_2_), were developed and tested as new consolidants for stone and stone-like substrates as well as alkaline reservoir for wood. Their performance as consolidants for indoor and outdoor applications on wall paintings was evaluated both in laboratory and in the field in comparison with commercial products and the results published in recent papers [15, 16]. In particular, short-term surface colour change induced by the application of the consolidation treatments was investigated in laboratory using colorimetric measurements carried out on wall paintings mock-ups. Besides the consolidants, other products are usually applied on wall paintings in order to prevent damage due to the growth of microorganisms [6, 17]. Hence, the innovative consolidants were also tested in combination with two commercial biocides, and the color changes due to the possible interaction between the consolidants and the biocides were evaluated. Due to the quite huge amount of data collected and the different sources of variability, it was difficult to draw general conclusions using traditional statistical analysis and visualization, as reported in a previous publication [16].

For this reason, in the present paper multivariate analysis techniques [18] were used to explore relationships between and trends among the selected treatments and their effect on the colour change of the wall painting samples taking into account both the pigments and the painting techniques. In fact, the use of multivariate data analysis allows a decomposition of this complex data into simpler structures, hence an easier and more effective interpretation of the results, and the possibility to take into account at the same time all the sources of data variability, i.e. treatments, painting techniques and pigments.

Therefore, Principal Component Analysis (PCA) [19] was carried out to explore data and to extract information concerning the effect of the commercial and innovative treatments on the different pigments in combination with the painting technique.

Being the data characterized by more than two sources of variability, Parallel Factor Analysis (PARAFAC) [20, 21] was then performed. PARAFAC is an extension of PCA to third or higher order data array, therefore it is particularly useful in case of data with multi-way nature. PARAFAC was applied in order to appreciably improve data visualization and interpretation, as well as to complete PCA results. In particular, as pigments are key features of painted surfaces, the effect of consolidation treatments on this specific variable is a crucial issue that has to be studied carefully.

Finally, PCA and PARAFAC results were compared to the ones obtained with a univariate analysis.

Despite the application of multivariate techniques in the field of conservation science is relatively recent [22], it shows an increasing trend [23, 24]. Several specific conservation issues can be addressed with multivariate approach; in particular, PCA has been successfully applied to explore and to classify data collected in studies of cultural artefacts [25, 26]. Moreover, PCA has been recently used to analyse colorimetric data from samples of archaeological and cultural interest [27, 28], nevertheless, to the best of the authors’ knowledge, this work is the first attempt to apply multi-way PARAFAC technique on colorimetric data collected on model samples of historic surfaces. In addition, the results of the colorimetric measurements performed on the combination of consolidant and biocide treatments are presented here for the first time.

## Experimental

### Samples and treatments

**Fig. 1:**
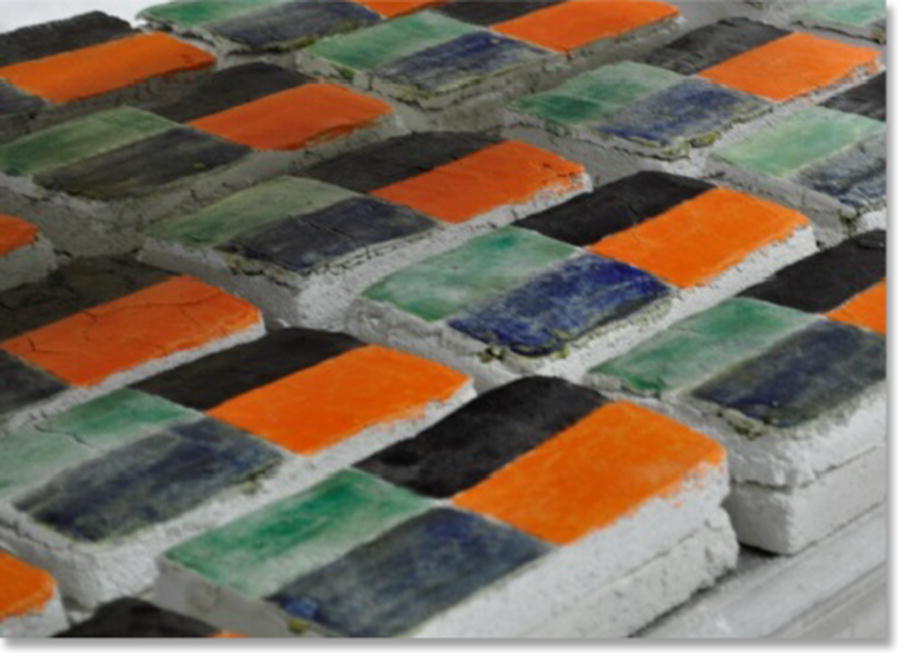
Wall painting mock-ups painted with orange minium, blue smalt, green malachite and carbon black (clockwise from top left of each specimen)

All the 20 × 10 × 5 cm wall paintings mock-ups were made of two lime mortar layers, a bottom coarse one and a top fine one, covered by a paint layer to replicate the structure of historic wall paintings, as described in detail in [16]. Three different painting techniques, which may influence the effect of a consolidant on a painted substrate, were used: (i) affresco (pigments are applied on fresh lime mortar without any binder) (named F in the text); (ii) pigments mixed with a polymerized linseed oil binder (O) (Les Établissements de peinture Grupp, Souffelweyersheim, France); (iii) pigments mixed with an egg yolk binder (E). Twelve pure pigments (Kremer pigmente, Aichstette, Germany) were tested: blue smalt (shorten as b for simplicity), carbon black (c), green malachite (g), orange minium (o), blue azurite (a), manganese black (m), Naples yellow (n), red vermilion (v), green earth (e), raw Sienna (s), red ochre (r) and yellow ochre (y). Four pure pigments were applied on each wall painting mock-ups in each of its four equal quarters (Fig. 1). In addition some wall painting mock-ups was kept unpainted. Detailed description of the mock-ups can be found elsewhere [16].

The two innovative and two commercial consolidants compared in the present study are listed in Table 1. A more detailed description of the new products is reported in [15, 16]. The consolidants were applied one time by brush on the surface of the wall painting mock-ups until apparent saturation, but for each pigment-painting technique combination, some mock-ups were kept untreated and were considered as reference.

**Table 1:** Consolidants and biocides tested on wall painting mock-ups

Name (acronym used in the present paper)	Chemical composition/manufacturer
Innovative products	
Ca(OTHF)_2_ (H)	Consolidant: hite solid, dissolved in 1:1 ethanol:ligroin at 20 g/L of Ca (ABCR, Spain)
Ca(OEt)_2_ (E)	Consolidant: nanosuspension in THF/EtOH, diluted with ethanol at 20 g/L of Ca (ABCR, Spain)
Commercial products	
Primal™ E 330 S (P)	Consolidant: acrylic emulsion in water applied pure as recommended by the manufacturer (CTS Srl, Paris, France)
CaLoSiL^®^ E50 (C)	Consolidant: nanodispersion of Ca(OH)_2_ in ethanol (IBZ-Salzchemie, Freiberg, Germany), with aninitial Ca concentration of 27.05 g/L, was diluted with ethanol until a Ca concentration of 20 g/L—the same chosen for alkoxides
Biotin T (B)	Biocide: contains didecyldimethylammonium chloride (large spectrum. Efficient against all types of micro-organisms) and 2-octyl-4-isothiazolin-3-one, a fungicide. Used diluted in distilled water at 3% v/v as recommended the distributor (CTS Srl, Paris, France)
Proxymousse (Py)	Biocide: contains benzododecinium chloride (2.5% w/w) (large spectrum biocide). Applied pure as recommended by the manufacturer (Peintures et chimie, Caudry, France)

For 8 of the 12 pigments, some of the painted mock-ups treated with the innovative consolidants received a second treatment with one of two commercial biocides containing quarternary ammonium compound as an active substance (Table 1). These were chosen because of their biocide activity towards algae and fungi, microorganisms usually found respectively in stone and wall paintings. The testing of a combination of a biocide and a consolidant was carried out to check if each product did not interact negatively with the other when used one after the other on the same surface, a situation that may arise in the field. For these mock-ups the consolidant was applied first, then after at least a week, the biocide was applied with a brush until saturation was reached, i.e. about 15 mL of biocide solution per mock-up. The mock-ups treated with both a consolidant and a biocide were painted with the following eight pigments: blue smalt, orange minium, carbon black, green malachite, blue azurite, red vermilion, manganese black and Naples yellow.

### Colorimetric measurements

Surface colour changes of the painted wall painting mock-up areas due to the application of the treatments were evaluated by colorimetric measurements with a Konica Minolta CM-2300d portable spectrophotometer. Measurements were acquired referring to the CIE L*a*b* [29] chromaticity diagram, and the ISO 11664-4:2008 [30] and UNI 8941-2/87 [31] standards. The standard illuminant D65/10° was used, including the specular reflection component, through a measuring field of 8 mm in diameter. The L*, a* and b* values were measured before and after the application of the treatments at three random locations on each of the four pigment quadrants of each mock-up surface, making sure that no crack was in the measurement spot. There were three identical mock-ups for each pigment-painting technique-treatment combination. Chroma, C*, was calculated for each location and for each mock-up using the measured value of a* and b*. The following differences between the situation after and before treatment were calculated for each location and each mock-up: ΔL*, Δa*, Δb*, ΔC*. Moreover, the ∆E*_ab_ index for total colour difference (∆E* in this study) was calculated with the formula [29]:

$$ \Delta E_{ab}^{*} = \sqrt {\left( {\Delta a^{*} } \right)^{2} + \left( {\Delta b^{*} } \right)^{2} + \left( {\Delta L^{*} } \right)^{2} } . $$

Then the 9 values of ∆L*, ∆a*,∆b*, ∆C* and ∆E* available for each pigment-painting technique-treatment combination were averaged and used for data analysis.

### Multivariate statistical analysis

In order to extract the relevant information and to fully analyse the different variability sources, i.e. consolidation treatments (with and without biocides), painting techniques and pigments, two explorative data analysis techniques were employed, namely PCA and PARAFAC. The principles of both methods are briefly recalled while for a more detailed description, the reader is referred to the relevant literature [19–21].

#### PCA analysis

Principal Component Analysis (PCA) is a dimension reduction method [19], used to capture the relevant information and to visualize major trends and structure of data. In particular, a set of orthogonal variables (called principal components, PCs) is generated as weighted linear combinations of the original variables (in the present case, represented by colorimetric parameters), following the model:

$$ x_{ij} = \sum\limits_{f = 1}^{F} {t_{if} } p_{jf}^{{}} + e_{ij}^{{}} $$

where *F* is the number of components used in the PCA model; **T** (*I *× *F*) with element *t*_*if*_ is the score matrix, expressing the coordinates of samples in the principal components space; **P** (*J *× *F*) with element *p*_*jf*_ is the loadings matrix expressing the weight of each original variable on a given principal component; e_ij_ is a residual term containing all the unexplained variation.

In total 240 sample areas were considered in this study: 144 sample areas coming from the application of 4 consolidant treatments on 12 pigments tested with 3 different painting techniques, and 96 sample areas from the application of 4 consolidant—biocide treatments on 8 pigments painted with the same 3 different painting techniques. PCA analysis was carried out in order to capture the variation/information held in all the 240 sample areas with the most dominant principal components. The data was arranged in a two-dimensional matrix (240 × 5 dimensions, 5 is the number of the colorimetric variables included in the analysis, i.e. ΔL*, Δa*, Δb*, ΔC* and ΔE*). No data pretreatment, such as mean centering and variance scaling [19], was applied since colour difference values were calculated a priori for each sample area with respect to an untreated reference sample area characterized by the same painting technique and the same pigment. The number of principal components to be retained was selected on the basis of the percentage of total explained variance, not to be lower than 90%. PCA was performed with the software PLS Toolbox 8.1 (Eigenvector Research, Inc., Wenatchee, WA, USA) for Matlab ©.

#### PARAFAC analysis

Parallel factor analysis (PARAFAC) is a generalization of PCA to higher order arrays [20, 21]. Mathematically, given a three-way array **X** of dimension I × J × K, with elements x_i,j,k_, it is decomposed as a sum of triple product of vectors and the PARAFAC model can be expressed as follows:

$$ x_{ijk} = \sum\limits_{f = 1}^{F} {a_{if} } b_{jf}^{{}} c_{kf}^{{}} + e_{ijk}^{{}} $$

where **A** (*I *× *F*) with element *a*_*if*_ is the first mode score matrix, **B** (*J *× *F*) with element *b*_*jf*_ and **C** (*K *× *F*) with element *c*_*kf*_, are the second and the third mode weights, respectively. *F* is the number of factors used in the PARAFAC model; e_ijk_ is a residual term containing all the unexplained variation.

The PARAFAC model provides parameters (loadings) that directly reflect the variability in the modes of interest (i.e. treatments, colorimetric parameters, painting techniques and pigments).

Thus, the variation in each mode is described by a low number of underlying latent phenomena.

In this study, since biocides were tested only on 8 pigments (instead of 12), two different data analysis were carried out (referred as All data analysis and Reduced data analysis, respectively): the first dataset including all the 12 pigments and excluding the biocides (i.e. only 4 of the 8 treatments), the second one including all the 8 treatments (4 with and 4 without biocides) and only the pigments (8) on which the biocides were tested. This procedure was applied in order to avoid problems due to missing data and to obtain as much as possible information on variation due to the biocides presence.

In both data analyses, 3-way arrays were built. The 3-way arrays reported the treatments in the first mode, the colorimetric parameters in the second mode, the painting technique and pigments in the third mode, i.e. (4 × 5 × 36 and 8 × 5 × 24 dimension arrays for the first and second data analyses, respectively). The choice to build 3-way arrays was due to the need to highlight a clear information about differences among pigments as well.

Also in this case, no data pretreatment was performed and PARAFAC analysis was carried out using PLS Toolbox 8.1 for Matlab ©.

For the choice of the right number of PARAFAC factors, several different criteria were evaluated, such as core consistency [21], percentage of explained variance and sum of squared errors.

## Results and discussion

An ideal treatment should not alter the visual appearance of the surface to which it is applied. In general, in the field of historic building conservation, a total colour difference (ΔE*) up to 5 units after a treatment application is generally considered unnoticeable to the human eye [32, 33].

**Fig. 2:**
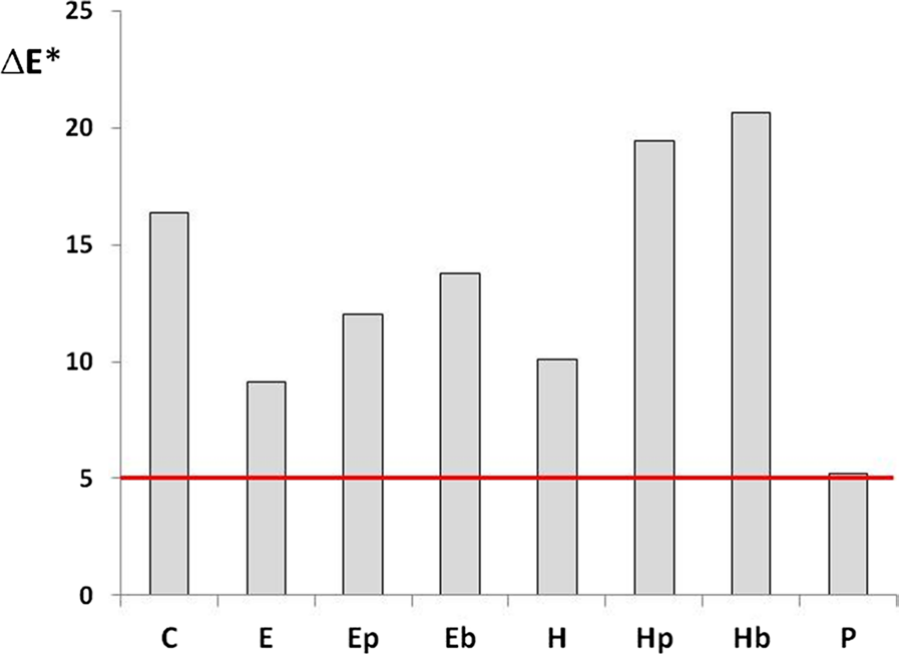
Average overall colour difference of wall painting mock-ups as function of treatments for all pigments and painting techniques. Meaning of the abbreviations is described in a list at the end of the article

The total colour difference after/before treatment exceeded the threshold value of 5 units for all the samples, except for the one treated with Primal E330 S (P) (Fig. 2). All the three consolidants that led to the formation of CaCO_3_, i.e. Ca(OTHF)_2_, Ca(OEt)_2_ and CaLoSiL, almost always induced much higher colour change than Primal E330 S, regardless of the painting technique and pigment. Moreover, the application of a biocide after the consolidant almost always increased ΔE*, regardless again, of the painting technique and pigment, the effect being generally slightly more notable for Biotin T (Hb and Eb vs respectively H and E) than for Proxymousse (Hp and Ep vs respectively H and E). This result can be explained by the fact that the presence of the consolidant could inhibit the penetration of the biocide which tended to stay on the sample surface and thus to increase the colour change. This effect was particularly remarkable for Ca(OTHF)_2_ and it seemed to be related to the consolidation efficacy of the alkoxide in terms of surface hardening, as laboratory tests indicated that a stone surface treated with Ca(OTHF)_2_ was more resistant than a one treated with Ca(OEt)_2_ [16].

Besides this general consideration, the univariate approach applied in a previous study [16] to the colorimetric data was quite consuming, as it required to consider by twos the different sources of data variability, moreover it was difficult to extract rational and relevant features related to the whole dataset. Finally, the high dispersion of the data related to some pigments made quite challenging the understanding of the variability related to the pigments themselves [16].

These problems were successfully overcome by the use of a multivariate approach, i.e. PCA and PARAFAC analyses, described in the following sections.

### PCA of colorimetric data

**Fig. 3:**
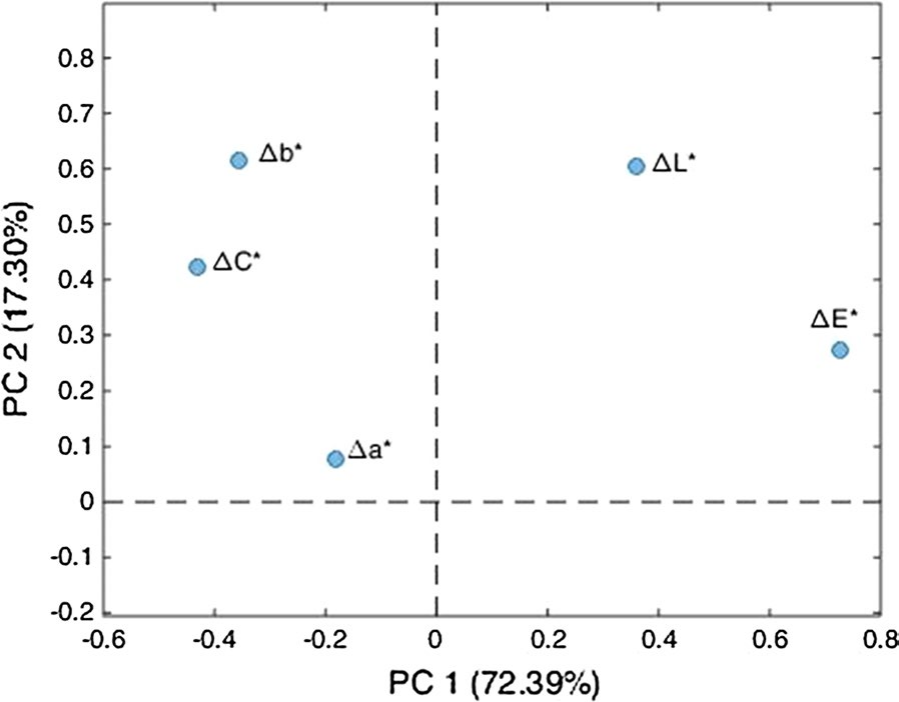
Loading plot of PC1 vs PC2 on the 240 × 5 dataset

**Fig. 4:**
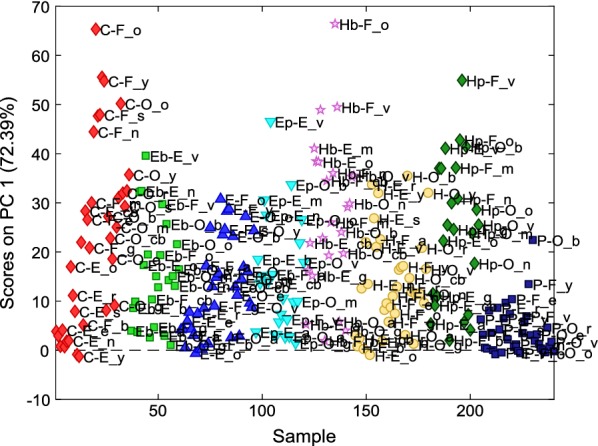
Score plot on PC1 of the 240 sample areas. The 8 treatments are represented in different colours: CaLoSiL (C) in red; Ca(OEt)_2_ + Biotin T (Eb) in light green; Ca(OEt)_2_ (E) in blue; Ca(OEt)_2_ + Proxymousse (Ep) in light blue; Ca(OTHF)_2_ + Biotin T (Hb) in pink; Ca(OTHF)_2_ (H) in yellow; Ca(OTHF)_2_ + Proxymousse (Hp) in dark green; Primal E 330 S (P) in dark blue. Meaning of the abbreviations related to painting techniques and pigments is described in a list at the end of the article

Figure 3 shows the loadings plot (colorimetric variables plot) of PC1 vs. PC2. The total variance accounted by the first two PCs was around 90%, therefore the discussion of the results is focused on PC1 and PC2 only. In particular, Fig. 4 visualizes the score plot of PC1, which is responsible alone to the description of 72% of total variance.

In Fig. 3, PC1 shows positive loadings for ΔE* and ΔL*, while negative loadings for ΔC* and Δb*. PC2 shows positive loadings for all the variables, in particular Δb* and ΔL*. Finally, Δa* is not relevant in any of the two first PCs, and Δb* and ΔC* appear to be directly correlated to each other.

Almost all samples are characterized by positive scores on PC1 (Fig. 4), hence, for most of them, after treatment ΔE* and ΔL* increase, whilst Δb* and ΔC* decrease. For all the pigment-painting technique combination, the samples treated with Primal E330 S are the most homogeneous, characterized by the lowest variation of the colorimetric variables. The samples treated with CaLoSiL (C), Ca(OTHF)_2_ + Biotin (Hb) and Ca(OTHF)_2_ + Proxymousse (Hp) show the highest ΔE* increase and ΔC* decrease. PC2 (data not shown) mainly set apart sample areas painted with technique O and pigment b and treated with Hp and Ep that are characterized by the highest increase of ΔL* and Δb*.

Anyhow, a general tendency of the effect of the different treatments can be observed regardless of the painting technique and pigment. Ca(OTHF)_2_, Ca(OEt)_2_ and CaLoSiL induces higher changes than Primal E330 S in almost all the colorimetric variables. In particular, the increase of ∆E* and decrease of ∆C* follow the scale: CaLoSiL (C) > Ca(OTHF)_2_ (H) ≥ Ca(OEt)_2_ (E) > Primal E330 S (P). Moreover, the addition of a biocide to the innovative consolidant treatments seems to have a remarkable effect on colour variation only when combined with Ca(OTHF)_2_. PCA confirms the general conclusions drawn by means of the univariate approach (Fig. 2) [16], speeding up and simplifying data analysis. Nevertheless, the dependence from the painting technique and from the pigment was not distinguishable with the two dimensional model, due to the quite huge amount of data, hence the multi-way PARAFAC method has been applied to deal with these sources of data variability.

### PARAFAC analysis of colorimetric data

In PARAFAC analysis, each source of variability constitutes a so-called ‘mode’ and the variation in each mode can be described by a low number of factors, improving and simplifying the visualization of the results.

### All data analysis results

The analysed dataset includes all the pigments but only the consolidant treatments with no biocide added.

One-factor model with an explained variance of 68% has been chosen for the 3-way array because of its high core consistency (100%) and its robustness considering the lowest values of the sum of the squared residuals.

**Fig. 5:**
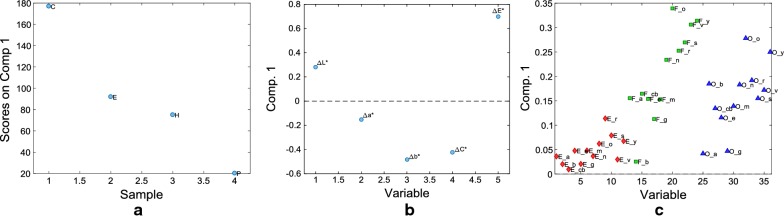
3-way PARAFAC model. Loadings on factor 1 of the three modes of all data analysis: **a** Mode1-treatments; **b** Mode2-colorimetric parameters; **c** Mode 3-painting techniques and pigments

The loading plots of the first (treatments), second (colorimetric parameters) and third modes (painting techniques × pigments) of the first factor are reported in Fig. 5.

In the first mode plot (Fig. 5a), all treatments have positive scores values. However, the first factor mainly differentiates treatment C (characterized by the highest scores value) from H, E and P. In particular, it is clear that the samples treated with CaLoSiL (C) are the ones characterized by the most remarkable increase of ∆E* and decrease of ∆C* (and ∆b*) (Fig. 5b). This behavior is particularly true for almost all samples painted with affresco (F) and oil blinder (O) techniques which seem to be directly correlated since they present two parallel trends for all the pigments. From an explorative point of view, Fig. 5c shows the presence of three groups: the first one includes samples with lower loading values, i.e. all samples painted with E technique regardless of the pigment, and three other samples: two samples painted using oil binder one with azurite and the other with green malachite (respectively O_a and O_g) and one sample painted blue smalt applied with affresco technique (F_b); the second group includes pigments o, y, v, s, r and n applied using affresco technique (F), and o and y mixed with oil binder (O); and the third one all the other pigments in the middle. The first factor clearly distinguishes samples from the first and second group, while some of the samples in the third group show a certain degree of overlap with the first group, in particular F_g and O_e. The colorimetric variation of samples F_b, O_a and O_g is similar to the one of all the pigments treated with E and, in particular, they present the lowest ∆E* variation among F and O painting technique samples, respectively. Indeed, the role of the painting technique appears quite clearly in the 3-way PARAFAC model, showing the samples painted with affresco technique or using oil binder more distributed than the ones with egg binder. This model allows to draw up some preliminary conclusions on the “pigment” variable: in fact, it seems that the pigments characterized by a higher colour saturation, e.g. orange minium, are the more prone to color changes due to the application of consolidants, especially CaLoSiL.

In particular, from a deeper analysis of Fig. 5c, the samples characterized by higher loadings on factor 1, i.e. samples belonging to group 3, ware more prone to an increase of ∆E* and decrease of ∆C*, after the application of the consolidants.

The results obtained with all data 3-way analysis are in complete agreement with the conclusions drawn using the traditional statistical data analysis and visualization [16].

### Reduced data analysis results

One-factor model with an explained variance of 60% has been chosen for the 3-way arrays considering the above mentioned criteria.

**Fig. 6:**
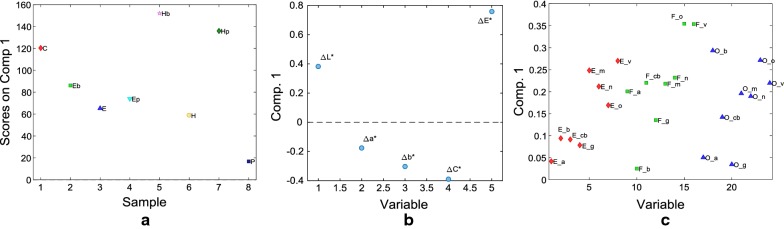
3-way PARAFAC model. Loadings on factor 1 of the three modes of reduced data analysis: **a** Mode 1-treatments; **b** Mode 2-colorimetric parameters; **c** Mode 3-painting techniques and pigments

The used dataset includes all the treatments, with and without biocides, and only the 8 pigments on which biocides were tested. From the analysis of the loadings on factor 1 of the 3-way PARAFAC model it is clear that the samples treated with CaLoSiL alone, and with Ca(OTHF)_2_ followed by Biotin T or Proxymousse are the ones characterized by the greatest increase of ΔE* and decrease of ΔC* (Δb*) (Fig. 6a, b). This is particularly true for the two samples realized with affresco technique F_o and F_v, respectively painted with orange minium and and red vermillion (yellow ochre was not tested with biocide) (Fig. 6c). As already pointed out, this result can be related to the consolidation efficacy of the Ca(OTHF)_2_ in terms of surface hardening, that for affresco technique is higher than for the other treatments [16]: the greater the consolidation effect, the lesser the penetration of the biocide in the substrate, the greater the surface color change. Similar differences with respect to the previous PARAFAC model, can be found among the colorimetric parameters, i.e. ΔE* and ΔL* are directly correlated with positive loadings and inversely correlated to ΔC*, Δb* and Δa* with negative loadings (Fig. 6b). Although, similar trend is still present between F and O techniques, the application of a biocide clearly increases the variation of colorimetric data of pigments v, m, n and o of samples painted with E technique (Fig. 6b, c). In addition, the behavior of pigments mixed with egg binder (E) aree not particularly homogeneous and they present very different loadings values (Fig. 6c). In particular, there is a clear difference between E_v, E_m, E_n and E_o (samples more similar to some samples made with the F and O painting techniques) and E_a, E_b, E_cb and E_g with lower loading values (samples more similar to F_b, O_a and O_g as in the previous PARAFAC model).

## Conclusions

The multivariate approach turns out to be very useful in the study of the compatibility of wall painting consolidants in terms of surface colour variation, simplifying the analysis of the huge amount of data collected and leading to an easier and more effective interpretation of the results when compared to the univariate approach.

In particular, the 3-way PARAFAC model provides a powerful technique to investigate at the same time the different sources of colorimetric data variability, i.e. treatments, painting techniques and pigments, outperforming the traditional statistical analysis in the study of the dependence of colour variation, especially on pigments, a critical issue for painted surfaces.

PCA and PARAFAC results proves that the two innovative calcium alkoxide consolidants induce less important colour variations on the wall paintings surfaces than the well-known CaLoSiL, but higher variation than the commercial Primal E 330 S. Moreover, the application of biocides after the alkoxides seems to enhance surface colour change, especially in case of Ca(OTHF)_2_. The overall colour variation induced by the alkoxide treatment (applied or not with biocide) is generally higher than the threshold accepted in the cultural heritage conservation field, but this effect can be related to the concentration of the products applied and also to their ageing, as already pointed out in [16].

The proposed multivariate approach can be applied to data sets from further laboratory tests, characterized by more sources of data variability, e.g. product concentration, type and amount of solvent, and so forth. In addition, multi-way techniques can be a useful method to explore data collected in situ where climatic conditions and exposure time might also play an important role in the colour change of treated surfaces.

Finally, even though the results indicates that Primal E330 S leads to the least overall color variation compared to the other consolidants tested, further investigations are required to confirm this tendency over time, as the yellowing of acrylic products due to ageing is a well-known phenomenon [34].

## Abbreviations

### Multivariate analysis

PCA: Principal Component Analysis
PC: Principal Component
PARAFAC: Parallel Factor Analysis

### Consolidation treatment

H: Ca(OTHF)_2_
E: Ca(OEt)_2_
C: CaLoSiL
P: Primal E 330 S

### Combination consolidant treatment/biocide

Hb: Ca(OTHF)_2_ + Biotin T
Hp: Ca(OTHF)_2_ + Proxymousse
Eb: Ca(OEt)_2_ + Biotin T
Ep: Ca(OEt)_2_ + Proxymousse

### Painting technique

F: affresco
O: oil binder
E: egg binder

### Pigment

b: blue smalt
c: carbon black
g: green malachite
o: orange minium
a: blue azurite
m: manganese black
n: Naples yellow
v: red vermilion
e: green earth
s: raw Sienna
r: red ochre
y: yellow ochre
